# FOXP3-microRNA-146-NF-κB as oncotarget

**DOI:** 10.18632/oncoscience.220

**Published:** 2015-08-26

**Authors:** Deepa M. Etikala, Runhua Liu, Lizhong Wang

**Affiliations:** University of Alabama at Birmingham, Birmingham, AL, USA

**Keywords:** FOXP3, microRNA146, NF-κB, breast cancer, prostate cancer, gene therapy

The X-linked FOXP3 gene is a member of the forkhead box/winged helix family. As a transcription factor, FOXP3 plays an important role in the immune system by acting as a master regulator of transcription for the development and function of regulatory T cells. Additionally, its tumor suppressive activity has been observed during tumor initiation [1]. MicroRNA (miR) plays a role in RNA silencing and in post transcriptional regulation of gene expression. Accumulating data suggest that the miR-146 family, including miR-146a/b, inhibits cancer cell proliferation, invasion and metastasis in human breast and prostate cancers [2-5]. Since miR-146a is overexpressed in Foxp3^+^ regulatory T cells and is critical for its function in the immune system, there is thought to be a link between miR-146a and the FOXP3 gene [6]. Additionally, nuclear factor-kappaB (NF-κB), a protein complex that controls transcriptional activity of DNA and helps regulate cell survival, was instrumental to the discovery of a novel pathway that may serve as a target of drug therapy for cancer patients.

**Figure 1:**
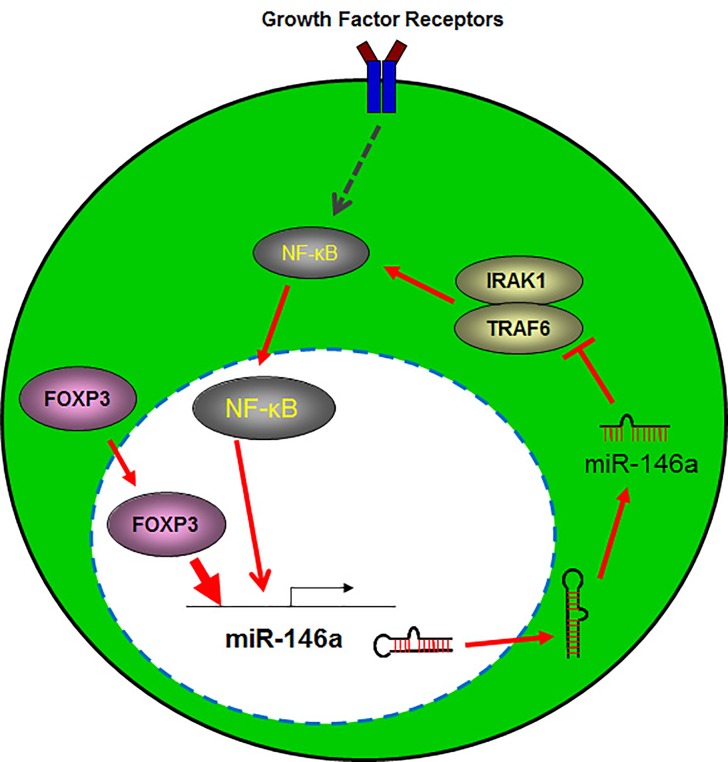
FOXP3 directly targets miR-146a and regulates the miR-146a and NF-κB feedback loop in cancer cells FOXP3 induces the expression of miR-146a, which plays a key role in the negative feedback regulation of NF-κB in cancer cells, thereby reducing the proliferation, invasion and metastasis of cancer cells. Furthermore, through the inhibition of the expression of two miR-146 target genes *IRAK1*, Interleukin-1 receptor-associated kinase 1 and *TRAF6,* TNF receptor associated factor 6, there is subsequent tumor suppression in cancer cells.

In these two papers, a new axis was identified and is known as the FOXP3-miR-146-NF-κB axis (Fig. 1) [7, 8]. In breast cancer cells, chromatin immunoprecipitation sequencing was used to identify a series of potential FOXP3-targeted miRs [7]. In particular, FOXP3 dramatically induced the expression of miR-146a/b that prevented tumor cell proliferation while also enhancing apoptosis. Functional analyses showed that FOXP3-induced miR-146a/b downregulated NF-κB activation by inhibiting the expression of two miR-146 target genes *IRAK1* (involved in Toll/IL-1 signaling) and *TRAF6* (a member of a family of proteins involved in the regulation of inflammation and apoptosis) [7]. Furthermore, chromatin immunoprecipitation assays revealed that FOXP3 directly bound to the promoter region of miR-146a but not to that of miR-146b. In addition, a direct interaction of FOXP3 with NF-κB p65 was identified to regulate a miR-146a-NF-κB negative feedback regulation loop in normal breast epithelial cells as well as in breast cancer cells (Figure 1). *In vivo* analyses also validated that FOXP3-mediated induction of miR-146a resulted in the downregulation of *IRAK*1 and *TRAF*6 and subsequently inhibited NF-κB activation, thus leading to tumor suppression in breast cancer cells. Conversely, FOXP3-mediated inhibition of cell proliferation and tumor growth and its induction of apoptosis were partially blocked by miR-146a/b inhibitors, indicating a contribution of miR-146a/b to FOXP3-triggered tumor suppression.

It has been determined that the FOXP3-miR-146-NF-κB axis serves as a target pathway for diminishing the pre-prostate cancerous state in the animal model [8]. In prostate cancer cells, it was also validated that FOXP3 transcriptionally inhibits *IRAK1* and *TRAF6* through an induced expression of miR-146a/b [8]. Hybridization analysis indicated that a low expression of miR-146a/b was observed in prostate cancer cell lines, while a high expression of miR-146a/b was seen in normal prostate tissues [4]. Transfection of miR-146a into prostate cancer cells resulted in a marked reduction of cell invasion, proliferation and metastasis to bone marrow [4, 5], suggesting that miR-146a functions as a tumor suppressor in prostate cancer cells. The functional FOXP3-miR-146-NF-κB axis during tumor initiation in prostate cancer cells was identified in prostate *Foxp3* conditional knockout (*Foxp3*cKO) mice. It was further noted that miR-146a KO mice and *Foxp3*cKO mice developed prostatic intraepithelial neoplasia (PIN, a pre-prostate cancerous state), but not prostate cancer, suggesting that miR-146a as well as FOXP3 have a tumor-suppressive role during tumor initiation [1, 8]. Since FOXP3 can functionally interact with NF-κB to induce transcriptional activity of miR-146a, the FOXP3-miR-146-NF-κB axis could be a potential therapeutic target for cancers with FOXP3 defects. In fact, the NF-κB inhibitor bortezomib induced apoptosis in prostate epithelial cells of *Foxp3*cKO mice [8], suggesting that NF-κB-related apoptosis is involved in FOXP3-mediated tumor suppression. Although bortezomib did not completely eliminate PIN formation, its administration inhibited mouse prostate tumor growth and reduced PIN incidences. This was most likely due to an increase in apoptotic cells and shows the potential target of the FOXP3-miR-146-NF-κB axis in patients experiencing a precancerous state.

Ultimately, FOXP3-induced miR-146a/b inhibited NF-κB activation by repressing *IRAK1* and *TRAF6*, which led to tumor suppression and apoptosis during tumor initiation in breast and prostate epithelial cells. Additionally, the identification of the FOXP3-miR-146-NF-κB axis provides a mechanism for NF-κB activation as well as miR-146a/b disruption in breast and prostate cancer cells, allowing for a new therapeutic target in FOXP3 defect-related cancers.
